# Rs420137, rs386360 and rs7763726 polymorphisms in fibronectin type III domain containing 1 are associated with susceptibility to coronary heart disease: Analysis in the Han population

**DOI:** 10.3389/fcvm.2022.964978

**Published:** 2022-10-06

**Authors:** Xiaodan He, Xuemei Li, Xiaoyan Du, Jianlun Han, Hui Zhang, Yan Zhu, Honghong Ma

**Affiliations:** Department of Cardiovasology, The First Hospital of Yulin, Yulin, China

**Keywords:** *FNDC1*, CHD, susceptibility, genetic polymorphism, SNPs

## Abstract

**Background:**

Numerous genetic studies have shown that genes are related to the pathogenesis of coronary heart disease (CHD). The main aim of this study was to confirm whether fibronectin type III domain containing 1 (*FNDC1*) polymorphisms correlate with the risk of CHD.

**Methods:**

In this study, in order to assess the association between three *FNDC1* single nucleotide polymorphisms (SNPs) and the risk of CHD, we conducted a case-control study involving 630 patients with CHD and 568 healthy controls using Agena MassARRAY (Agena Bioscience, San Diego, CA, USA). Genotype distribution in case and control groups was analyzed by Chi square test. Odds ratios (ORs) and 95% confidence intervals (CIs) were calculated by logistic regression models adjusted for age, sex, smoking, and alcohol consumption to assess the correlation between SNPs and CHD risk.

**Results:**

Our results indicated that *FNDC1*-rs420137, -rs386360, and -rs7763726 played important roles in enhancing the risk of CHD. Subgroup analysis revealed that rs420137 increased the susceptibility to CHD in males, smokers, and patients aged ≤62 years. Rs360 had an increased risk of CHD in males, patients at aged ≤62 years, smokers, and non-drinkers. Furthermore, the association of rs7763726 with increased CHD risk was also observed in males, patients aged ≤62 years, smokers, and drinkers. Last but not least, these three SNPs we selected were protective factors against hypertension in CHD individuals.

**Conclusion:**

Our research suggest that *FNDC1*-rs420137, -rs386360, and -rs7763726 variants may be regarded as novel biomarkers for predicting CHD risk and other specific mechanisms of action of CHD need to be further studied.

## Introduction

Coronary heart disease (CHD) is a very common cardiovascular disease. It is estimated that the total number of deaths from cardiovascular disease is expected to increase to 23.3 million by 2030 (1). Since the 1970 s, CHD has been the main cause of death all over the world. In China, 700,000 people die of CHD annually (2). In addition, the morbidity of CHD is rising. The occurrence and development of CHD correlate with a variety of environmental factors, including social factors (aging, employment, income, and education), behavioral factors (dietary habits, drinking, smoking, and exercise), and metabolic factors obesity, diabetes mellitus (DM), triglyceride (TG), and high-density lipoprotein cholesterol (HDL-C) (3). However, an increasing number of studies have shown that genetic factors play a critical role in the progression of CHD. By far, more than 60 genetic variants have been identified to be related to an increased susceptibility to CHD in genome-wide association study (GWAS) analyses (4). Interestingly, studies have shown that single nucleotide polymorphisms (SNPs) can affect the level of gene production or the binding of targets, thus influencing the normal expression of genes and leading to the occurrence of diseases (5, 6).

Fibronectin type III domain containing 1 (*FNDC1*) is a protein-encoding and disease-related gene located in the 6q25.3 region of the human chromosome and contains 102,713 bases, with a length of ~1,894 and a molecular weight of 205.6 kDa (7, 8). Moreover, *FNDC1*, also known as activator of G-protein signaling 8 (AGS8), is a positive factor in modulating the signal transduction system. It is expressed in various types of tissues, including thyroid, heart, fetal cartilage, adipose, and kidney tissues (9–11). As an important extracellular matrix protein, *FNDC1* plays a regulatory role in tumorigenesis and participates in cell migration, proliferation, and apoptosis. It is also involved in the pathogenesis of multiple blood and cardiovascular diseases (12). Previous studies have also shown that *FNDC1* has a certain relationship with hypertension (13).

To our knowledge, the effects of *FNDC1* gene polymorphisms on CHD have not been studied. Therefore, the present study explored the linkage between *FNDC1* polymorphisms and CHD susceptibility, which has considerable implications for the diagnosis and treatment of CHD.

## Materials and methods

### Study population

A total of 630 CHD cases and 568 controls were recruited from the First Hospital of Yulin City. All patients were independently judged by at least two cardiologists and diagnosed as CHD according to blood examination, coronary angiography, standardized electrocardiogram (ECG), and echocardiography. Out-patients in the control group were from the same hospital during the same period as the cases. According to previous medical history and angiographic diagnosis, they had no CHD, no obvious ischemic changes on ECG and no symptoms of chest pain. Participants with other heart diseases (rheumatic heart disease, cardiomyopathy, concomitant heart valve disease, or congenital heart disease) were eliminated from the study. Furthermore, it should be noted that patients with a history of intravenous thrombolysis, coronary stenting, angioplasty bypass surgery, or coronary artery disease were also excluded. Moreover, clinical indicators and basic characteristics of subjects were collected from medical records.

### DNA extraction

We collected 5 mL of fasting peripheral venous blood from each participant and put it in a test tube containing ethylene diamine tetraacetic acid (EDTA). According to the manufacturer's instructions, we extracted DNA from the collected blood samples using GoldMag-Mini whole blood genomic DNA purification kits (GoldMag Co., Ltd., Xi'an City, China).

### Genotyping of SNPs

First of all, three variants of FNDC1 (rs420137, rs386360, and rs7763726) were selected from the HapMap database according to the minor allele frequency (MAF) >5% and the unbalanced *r*^2^ value >0.8. Then, we genotyped them by the Agena MassARRAY platform (Agena Bioscience, San Diego, CA, USA). Finally, the results of genotyping were processed and analyzed by Agena Bioscience Typer software (version 4.0).

### Data analysis

The basic statistical analysis was conducted by Microsoft Excel (Microsoft Corp., Redmond, WA, USA) and SPSS 20.0 software (SPSS, Chicago, IL, USA). The *t*-test was used to assess continuous variables, while the Chi-square test was utilized to access categorical variables between samples. Meanwhile, the Chi-square test was used to evaluate whether the genotype distribution of these SNPs in the control group was in accordance with Hardy-Weinberg equilibrium (HWE), and the differences in the genotype and allele frequencies of these SNPs in two groups were also analyzed. PLINK 1.9 software (version 1.07) and Haploview 4.2 software were applied to analyze the association of genetic variants with CHD susceptibility under five genetic models, and odds ratios (ORs) and 95% confidence intervals (CIs) were calculated to confirm differences in this study. Besides, multifactor dimensionality reduction (MDR) was used to analyze the effects of SNP-SNP interactions on CHD susceptibility. Finally, a *p*-value < 0.05 indicated a statistical difference.

## Results

### Characteristics of subjects

The information on CHD patients is presented in Table 1. Patients with an average age of 62.16 ± 10.37 years included 429 males and 201 females, while controls with an average age of 61.46 ± 8.96 years included 360 males and 208 females. There was no statistically significant difference in the distribution of sex and age between the two groups (*p* > 0.05). In addition, the levels of HDL-C, TG, urea, alanine amino transferase (ALT), and blood glucose (BG) had no significant differences (*p* > 0.05) between controls and cases (Table 1). Nevertheless, other indicators [white blood cell count (WBC), red blood cell count (RBC), low-density lipoprotein cholesterol (LDL-C), platelet (PLT), hemoglobin (Hgb), total cholesterol (TC), absolute value of lymphocytes (LYMP), absolute value of neutrophils (NEUT), creatinine, and aspartate aminotransferase (AST)] were significantly different (*p* < 0.05) between the case and control groups (Table 1).

**Table 1:** Characteristic of patients with CHD and controls.

**Variables**	**Cases** **(*N* = 630)**	**Controls** **(*N* = 568)**	***P***
**Age, years (mean ± SD)**	62.16 ± 10.37	61.46 ± 8.96	0.252
>62 years	305 (48.4%)	289 (50.9%)	
≤62 years	325 (51.6%)	279 (49.1%)	
**Gender**			0.948
Male	429 (68.1%)	360 (63.4%)	
Female	201 (31.9%)	208 (36.6%)	
**Smoking**			
Yes	330 (52.4%)	260 (45.8%)	
No	300 (47.6%)	308 (54.2%)	
**Drinking**			
Yes	293 (46.5%)	266 (46.8%)	
No	337 (53.5%)	302 (53.2%)	
**Complicating hypertension**			
Yes	397 (63.0%)	152 (50.0%)	
No	233 (37.0%)	152 (50.0%)	
**Complicated with diabetes**			
Yes	180 (28.6%)		
No	450 (71.4%)		
WBC	6.82 ± 2.04	5.83 ± 1.47	**<0.001**
RBC	4.43 ± 1.99	56.5 ± 10.5	**<0.001**
HDL-C (mmol/L)	1.10 ± 0.26	1.13 ± 0.23	0.100
LDL-C (mmol/L)	2.40 ± 0.89	2.61 ± 0.74	**<0.001**
PLT (10^9^/L)	194.00 ± 56.87	213.26 ± 59.85	**<0.001**
HGB	131.10 ± 27.44	146.69 ± 14.34	**<0.001**
TG (mmol/L)	1.62 ± 1.00	1.75 ± 1.47	0.075
TC (mmol/L)	4.04 ± 1.08	4.75 ± 0.98	**<0.001**
Urea	5.26 ± 2.05	5.20 ± 1.23	0.513
LYMP (10E^10^/L)	8.06 ± 11.76	5.83 ± 1.47	**<0.001**
NEUT (10E^9^/L)	22.4 ± 28.43	7.55 ± 2.94	**<0.001**
Creatinine (μmol/L)	82.40 ± 20.97	68.26 ± 13.15	**<0.001**
AST	29.37 ± 33.53	25.59 ± 18.77	**0.009**
ALT	25.73 ± 22.03	25.63 ± 18.47	0.957
BG (mmol/L)	5.96 ± 2.61	6.06 ± 1.63	0.459

SD, standard deviation; WBC, white blood cell count; RBC, red blood cell count; HDL-C, high-density lipoprotein cholesterol; LDL-C, low-density lipoprotein cholesterol; PLT, platelet; HGB, hemoglobin; TG, triglyceride; TC, total cholesterol; LYMP, lymphocyte absolute value; NEUT, neutrophil absolute value; AST, aspartate aminotransferase; ALT, alanine aminotransferase; BG, blood glucose. Bold values are statistically significant (*p* <0.05).

### Association between *FNDC1* polymorphisms and CHD risk

Basic information on three SNPs (rs420137, rs386360, and rs7763726) in *FNDC1* is displayed in Table 2. All SNPs in the control group were in line with HWE (*p* > 0.05) and could be further analyzed. At the same time, we found that the frequency of the rs420137-G allele in the case group was significantly higher than that in the control group (*p* = 0.013, OR: 1.25, 95% CI: 1.05–1.48). The “C” allele of rs386360 (*p* = 0.010, OR: 1.26, 95% CI: 1.05–1.48) and the “A” allele of rs7763726 (*p* = 0.001, OR: 1.36, 95% CI: 1.13–1.64) were obviously related to an increased susceptibility to CHD.

**Table 2:** Basic information of SNPs in *FNDC1* gene.

**SNP ID**	**Gene**	**Chr: position**	**Allele**	**MAF**		**HWE**	**OR**	***P***
			**(minor/major)**	**Case**	**Control**	***p***	**(95% CI)**	
rs420137	FNDC1	6: 159231899	G / C	0.330	0.283	0.836	1.25 (1.05–1.48)	**0.013**
rs386360	FNDC1	6: 159239847	C / A	0.316	0.269	0.671	1.26 (1.05–1.50)	**0.010**
rs7763726	FNDC1	6:159249068	G / A	0.281	0.223	0.629	1.36 (1.13–1.64)	**0.001**

SNP, single nucleotide polymorphism; MAF, minor allele frequency; HWE, Hardy-Weinberg equilibrium; OR, odds ratio; 95% CI, 95% confidence interval.

*P*-values were calculated from χ^2^ test.

Bold values are statistically significant (*p* < 0.05).

As can be seen from Table 3, logistic regression analysis revealed that rs420137-GC was correlated with an increased risk of CHD under the co-dominant (*p* = 0.037, OR: 1.29, 95% CI: 1.02–1.65), dominant (*p* = 0.015, OR: 1.34, 95% CI: 1.06–1.68), and additive models (*p* = 0.011, OR: 1.27, 95% CI: 1.06–1.51). Rs386360-CA had a strong linkage with a higher susceptibility to CHD (co-dominant model: *p* = 0.026, OR: 1.31, 95% CI: 1.03–1.67; dominant model: *p* = 0.011, OR: 1.35, 95% CI: 1.07–1.70; additive model: *p* = 0.009, OR: 1.28, 95% CI: 1.07–1.53). It also showed that rs7763726-GA elevated CHD risk (co-dominant model: *p* = 0.001, OR: 1.50, 95% CI: 1.17–1.90; dominant model: *p* = 4.0E-04, OR: 1.52, 95% CI: 1.21–1.92; additive model: *p* = 7.0E-04, OR: 1.39, 95% CI: 1.15–1.69).

**Table 3:** Genotypes frequencies distribution and association with CHD risk.

**SNP-ID**	**Model**	**Genotype**	**Case (%)**	**Control (%)**	**OR (95% CI)**	***P***	**Adjusted OR (95% CI)**	***P***
rs420137	Co-dominant	CC	290 (51.1)	279 (44.3)	1		1	
		GC	234 (41.2)	286 (45.4)	1.27 (1.00–1.61)	0.049	1.29 (1.02–1.65)	**0.037**
		GG	44 (7.8)	65 (10.3)	1.54 (1.01–2.33)		1.55 (1.02–2.36)	
	Dominant	CC	290 (51.1)	279 (44.3)	1		1	
		GC-GG	278 (48.9)	351 (55.7)	1.31 (1.05–1.65)	0.019	1.34 (1.06–1.68)	**0.015**
	Recessive	CC-GC	524 (92.2)	565 (89.7)	1		1	
		GG	44 (7.8)	65 (10.3)	1.37 (0.92–2.05)	0.124	1.38 (0.92–2.06)	0.132
	Additive	–	–	–	1.04 (0.65–1.68)	0.013	1.27 (1.06–1.51)	**0.011**
rs386360	Co-dominant	AA	301 (53)	289 (45.9)	1		1	
		CA	228 (40.1)	283 (44.9)	1.29 (1.02–1.64)	0.035	1.31 (1.03–1.67)	**0.026**
		CC	39 (6.9)	58 (9.2)	1.55 (1.00–2.40)		1.56 (1.01–2.43)	
	Dominant	AA	301 (53)	289 (45.9)	1		1	
		CA-CC	267 (47)	341 (54.1)	1.33 (1.06–1.67)	0.014	1.35 (1.07–1.70)	**0.011**
	Recessive	AA-CA	529 (93.1)	572 (90.8)	1		1	
		CC	39 (6.9)	58 (9.2)	1.38 (0.90–2.10)	0.140	1.38 (0.90–2.11)	0.149
	Additive	–	–	–	1.27 (1.06–1.52)	0.010	1.28 (1.07–1.53)	**0.009**
rs7763726	C-odominant	AA	345 (60.7)	321 (51)	1		1	
		GA	193 (34)	264 (41.9)	1.47 (1.16–1.87)	0.002	1.50 (1.17–1.90)	**0.001**
		GG	30 (5.3)	45 (7.1)	1.61 (0.99–2.62)		1.68 (1.03–2.74)	
	Dominant	AA	345 (60.7)	321 (51)	1		1	
		GA-GG	223 (39.3)	309 (49)	1.49 (1.18–1.87)	7.0E-04	1.52 (1.21–1.92)	**4.0E-04**
	Recessive	AA-GA	538 (94.7)	585 (92.9)	1		1	
		GG	30 (5.3)	45 (7.1)	1.38 (0.86–2.22)	0.186	1.43 (0.88–2.31)	0.176
	Additive	–	–	–	1.37 (1.13–1.65)	0.001	1.39 (1.15–1.69)	**7.0E-04**

CHD, coronary heart disease; SNP, single nucleotide polymorphism; OR, odds ratio; CI, confidence interval. Adjusted OR (95% CI) were calculated by logistic regression analysis with adjustments for age and gender. Bold values are statistically significant (*p* < 0.05).

### Subgroup analysis of the correlation between *FNDC1* polymorphisms and CHD risk

We also performed stratified analyses of the association of three SNPs with CHD risk according to sex, age, smoking, drinking, hypertension, and diabetes to determine whether traditional risk factors influence the role of genetic variants in CHD. According to Table 4, we found that all variants increased the incidence of CHD under the allele, co-dominant, dominant, and additive models in males (rs420137: *p* = 0.006, 0.010, 0.004, and 0.006, respectively; rs386360: *p* = 0.007, 0.017, 0.006, and 0.006, respectively; rs7763726: *p* < 0.001, 0.001, 0.001, and 0.001, respectively). Rs420137 correlated with CHD risk in the allele (*p* = 0.047, OR: 1.29, 95% CI: 1.00–1.65), co-dominant (GG vs. CC *p* = 0.026, OR: 1.83, 95% CI: 1.01–3.31), recessive (*p* = 0.043, OR: 1.68, 95% CI: 0.95–2.99), and additive (*p* = 0.031, OR: 1.29, 95% CI: 1.00–1.65) models in individuals aged 62 years and younger.

**Table 4:** *FNDC1* SNPs associated with CHD risk stratified by gender and age status.

**SNP-ID**	**Model**	**Genotype**	**Male**		**Female**		>**62**		≤**62**	
			**OR (95% CI)**	***P***	**OR (95% CI)**	***P***	**OR (95% CI)**	***P***	**OR (95% CI)**	***P***
rs420137	Allele	C	1		1		1		1	
		G	1.36 (0.11–1.09)	**0.006**	1.01 (0.81–1.46)	0.573	1.21 (0.95–1.55)	0.121	1.29 (1.00–1.65)	**0.047**
	Co-dominant	CC	1		1		1		1	
		GC	1.48 (1.10–1.99)	**0.010**	1.00 (0.66–1.50)	0.997	1.37 (0.98–1.92)	0.142	1.20 (0.85–1.69)	0.300
		GG	1.65 (1.00–2.74)		1.38 (0.66–2.90)		1.32 (0.72–2.39)		1.83 (1.01–3.31)	**0.026**
	Dominant	CC	1		1		1		1	
		GC-GG	1.51 (1.14–2.00)	**0.004**	1.05 (0.71–1.55)	0.814	1.36 (0.98–1.88)	0.125	1.30 (0.94–1.80)	0.100
	Recessive	CC-GC	1		1		1		1	
		GG	1.38 (0.85–2.25)	0.194	1.38 (0.68–2.82)	0.372	1.12 (0.63–1.99)	0.649	1.68 (0.95–2.99)	**0.043**
	Additive	–	1.36 (1.09–1.69)	**0.006**	1.10 (0.80–1.50)	0.562	1.23 (0.96–1.59)	0.162	1.29 (1.00–1.65)	**0.031**
rs386360	Allele	A	1		1		1		1	
		C	1.36 (1.09–1.69)	**0.007**	1.11 (0.83–1.50)	0.498	1.24 (0.96–1.58)		1.28 (0.13–1.00)	0.051
	Co-dominant	A/A	1		1		1		1	
		C/A	1.44 (1.06–1.93)	**0.017**	1.12 (0.75–1.68)	0.502	1.39 (0.99–1.95)	0.124	1.22 (0.87–1.72)	0.279
		C/C	1.73 (1.01–2.94)		1.31 (0.60–2.84)		1.35 (0.72–2.52)		1.79 (0.96–3.33)	**0.027**
	Dominant	A/A	1		1		1		1	
		C/A-C/C	1.48 (1.12–1.96)	**0.006**	1.14 (0.77–1.69)	0.508	1.38 (1.00–1.91)	0.105	1.30 (0.94–1.80)	0.095
	Recessive	A/A-C/A	1		1		1		1	
		C/C	1.47 (0.88–2.47)	0.142	1.23 (0.58–2.61)	0.584	1.15 (0.63–2.11)	0.601	1.64 (0.89–3.00)	**0.044**
	Additive	–	1.37 (1.09–1.70)	**0.006**	1.13 (0.82–1.55)	0.445	1.26 (0.97–1.63)	0.131	1.29 (1.00–1.66)	**0.030**
rs7763726	Allele	A	1		1		1		1	
		G	1.58 (0.12–1.25)	**< 0.001**	1.09 (0.80–1.48)	0.287	1.33 (1.03–1.73)	**0.031**	1.40 (0.14–1.08)	**0.013**
	Co-dominant	A/A	1		1		1		1	
		A/G	1.87 (1.38–2.53)	**< 0.001**	0.98 (0.65–1.48)	0.337	1.45 (1.04–2.04)	0.088	1.52 (1.08–2.15)	**0.020**
		G/G	1.78 (0.96–3.29)		1.48 (0.66–3.30)		1.61 (0.80–3.24)		1.75 (0.88–3.49)	
	Dominant	A/A	1		1		1		1	
		G/A-G/G	1.86 (1.39–2.48)	**< 0.001**	1.04 (0.71–1.54)	0.831	1.47 (1.06–2.04)	0.055	1.55 (1.12–2.16)	**0.009**
	Recessive	A/A-G/A	1		1		1		1	
		G/G	1.39 (0.76–2.56)	0.283	1.49 (0.68–3.26)	0.317	1.37 (1.00–1.93)	0.316	1.49 (0.76–2.94)	0.182
	Additive	–	1.59 (1.25–2.03)	**< 0.001**	1.10 (0.80–1.50)	0.562	1.36 (1.04–1.78)	0.050	1.42 (1.09–1.86)	**0.008**

CHD, coronary heart disease; SNP, single nucleotide polymorphism; OR, odds ratio; CI, confidence interval.

OR (95% CI) values were calculated by logistic regression analysis with adjustments for gender and age.

Bold values are statistically significant (*p* < 0.05).

Table 4 also shows that rs386360 had a higher risk of CHD under the co-dominant, recessive, and additive models (*p* = 0.027, 0.044, and 0.030, respectively) in patients aged ≤62 years. Rs7763726 had an increased risk of CHD in patients aged ≤62 years in the allele (*p* = 0.013, OR: 1.40, 95% CI: 0.14–1.08), co-dominant (*p* = 0.020, OR: 1.52, 95% CI: 1.08–2.15), dominant (*p* = 0.009, OR: 1.55, 95% CI: 1.12–2.16), and additive models (*p* = 0.008, OR: 1.42, 95% CI: 1.09–1.86).

Stratified analyses by smoking and drinking (Table 5) demonstrated that rs420137 was significantly related to a higher risk of CHD under the allele model in smokers (*p* = 0.006) and drinkers (*p* = 0.044), and under the dominant (*p* = 0.017), recessive (*p* = 0.036), and additive models (*p* = 0.006) in smokers. Rs386360 increased the risk of CHD in smokers (allele model: *p* = 0.004, OR:1.46, 95% CI: 0.13–1.13; co-dominant model with the “CA vs. AA” genotype: *p* = 0.045, OR: 1.43, 95% CI: 1.00–2.02; dominant model: *p* = 0.010, OR: 1.55, 95% CI: 1.11–2.16; recessive model: *p* = 0.032, OR: 2.08, 95% CI: 1.06–4.08; additive model: *p* = 0.003, OR: 1.49, 95% CI: 1.14–1.95) and non-drinkers (co-dominant model with the “CA vs. AA” genotype: *p* = 0.027, OR: 1.45, 95% CI: 1.04–2.01; dominant model: *p* = 0.028, OR: 1.43, 95% CI: 1.04–1.95). In addition, rs7763726 enhanced the incidence of CHD in smokers (allele model: *p* < 0.001, OR: 1.83, 95% CI: 0.14–1.38; co-dominant model with the “AG vs. AA” genotype: *p* < 0.001, OR: 2.13, 95% CI: 1.48–3.05; dominant model: *p* < 0.001, OR: 2.19, 95% CI: 1.55–3.10; additive model: *p* < 0.001, OR: 1.91, 95% CI: 1.42–2.57) and drinkers (allele model: *p* = 0.002, OR: 1.56, 95% CI: 1.18–2.06; co-dominant model with the “AG vs. AA” genotype: *p* = 0.002, OR: 1.75, 95% CI: 1.22–2.51; dominant model: *p* = 0.001, OR: 1.75, 95% CI: 1.24–2.46; additive model: *p* = 0.003, OR: 1.51, 95% CI: 1.15–1.98). Meanwhile, in Table 6, we found that rs420137, rs386360, and rs7763726 were protective factors against the occurrence of hypertension in CHD patients (rs420137: allele model: *p* = 0.004, OR: 0.70, 95% CI: 0.55–0.89; co-dominant model with the “GC” genotype: *p* < 0.001, OR: 0.53, 95% CI: 0.38–0.76; dominant model: *p* < 0.001, OR: 0.54, 95% CI: 0.38–0.76; additive model: *p* = 0.002, OR: 0.68, 95% CI: 0.53–0.87; rs386360: allele model: *p* = 0.005, OR: 0.71, 95% CI: 0.55–0.90; co-dominant model: *p* = 0.003, OR: 0.59, 95% CI: 0.41–0.83; dominant model: *p* = 0.001, OR: 0.58, 95% CI: 0.41–0.81; additive model: *p* = 0.002, OR: 0.67, 95% CI: 0.52–0.87; rs7763726: allele model: *p* = 0.004, OR: 0.69, 95% CI: 0.54–0.89; co-dominant model: *p* = 0.001, OR: 0.56, 95% CI: 0.40–0.79; dominant model: *p* = 0.001, OR: 0.57, 95% CI: 0.41–0.79; additive model: *p* = 0.002, OR: 0.67, 95% CI: 0.51–0.87). Nevertheless, after stratified by diabetes, no other SNP except rs7763726 was found in the recessive model (*p* = 0.011, OR: 0.29, 95% CI: 0.11–0.75) to reduce the risk of diabetes in CHD individuals (Table 6).

**Table 5:** *FNDC1* SNPs associated with CHD risk stratified by smoking and drinking statues.

**SNP-ID**	**Model**	**Genotype**	**Smokers**		**Non-smokers**		**Drinkers**		**Non-drinkers**	
			**OR (95% CI)**	***P***	**OR (95% CI)**	***P***	**OR (95% CI)**	***P***	**OR (95% CI)**	***P***
rs420137	Allele	C	1		1		1		1	
		G	1.43 (1.10–1.84)	**0.006**	1.12 (0.12–0.88)	0.353	1.30 (1.01–1.69)	**0.044**	1.20 (0.95–1.52)	0.133
	Co-dominant	CC	1		1		1		1	
		GC	1.38 (0.97–1.95)	0.072	1.18 (0.84–1.65)	0.333	1.19 (0.84–1.69)	0.336	1.37 (0.99–1.91)	0.060
		GG	2.24 (1.18–4.25)		1.13 (0.64–2.02)		1.79 (0.98–3.26)		1.28 (0.70–2.28)	
	Dominant	CC	1		1		1		1	
		GC-GG	1.50 (1.07–2.09)	**0.017**	1.17 (0.85–1.61)	0.333	1.29 (0.92–1.90)	0.139	1.35 (0.99–1.86)	0.060
	Recessive	CC-GC	1		1		1		1	
		GG	1.94 (1.04–3.61)	**0.036**	1.04 (0.60–1.81)	0.883	1.66 (0.93–2.95)	0.086	1.08 (0.61–1.91)	0.785
	Additive	–	1.44 (1.11–1.88)	**0.006**	1.11 (0.86–1.42)	0.416	1.28 (0.99–1.65)	0.058	1.23 (0.95–1.57)	0.111
rs386360	Allele	A	1		1		1		1	
		C	1.46 (0.13–1.13)	**0.004**	1.11 (0.12–0.87)	0.417	1.27 (0.13–0.97)	0.084	1.25 (0.12–0.98)	0.069
	Co-dominant	A/A	1		1		1		1	
		C/A	1.43 (1.00–2.02)	**0.045**	1.19 (0.85–1.66)	0.320	1.15 (0.81–1.64)	0.428	1.45 (1.04–2.01)	**0.027**
		C/C	2.44 (1.22–4.87)		1.08 (0.60–1.97)		1.76 (0.93–3.34)		1.31 (0.71–2.40)	
	Dominant	A/A	1		1		1		1	
		C/A-C/C	1.55 (1.11–2.16)	**0.010**	1.17 (0.85–1.61)	0.342	1.24 (0.89–1.73)	0.211	1.43 (1.04–1.95)	**0.028**
	Recessive	A/A-C/A	1		1		1		1	
		C/C	2.08 (1.06–4.08)	**0.032**	0.99 (0.56–1.78)	0.997	1.65 (0.89–3.07)	0.112	1.09 (0.61–1.97)	0.764
	Additive	–	1.49 (1.14–1.95)	**0.003**	1.10 (0.86–1.41)	0.459	1.25 (0.96–1.62)	0.097	1.27 (0.99–1.64)	0.061
rs7763726	Allele	A	1		1		1		1	
		G	1.83 (0.14–1.38)	**< 0.001**	0.53 (0.13–0.85)	0.478	1.56 (1.18–2.06)	**0.002**	1.22 (0.95–1.57)	0.126
	Co-dominant	A/A	1		1		1		1	
		A/G	2.13 (1.48–3.05)	**< 0.001**	1.11 (0.80–1.56)	0.528	1.75 (1.22–2.51)	**0.002**	1.28 (0.93–1.78)	0.133
		G/G	2.66 (1.19–5.98)		1.12 (0.59–2.12)		1.76 (0.90–3.43)		1.43 (0.69–2.94)	
	Dominant	A/A	1		1		1		1	
		G/A-G/G	2.19 (1.55–3.10)	**< 0.001**	1.12 (0.81–1.54)	0.507	1.75 (1.24–2.46)	**0.001**	1.30 (0.95–1.78)	0.104
	Recessive	A/A-G/A	1		1		1		1	
		G/G	2.02 (0.91–4.47)	0.085	1.07 (0.57–1.99)	0.838	1.42 (0.74–2.74)	0.288	1.28 (0.63–2.60)	0.493
	Additive	–	1.91 (1.42–2.57)	**< 0.001**	1.08 (0.84–1.40)	0.539	1.51 (1.15–1.98)	**0.003**	1.24 (0.95–1.62)	0.106

CHD, coronary heart disease; SNP, single nucleotide polymorphism; OR, odds ratio; CI, confidence interval. OR (95% CI) values were calculated by logistic regression analysis with adjustments for gender. Bold values are statistically significant (*p* < 0.05).

**Table 6:** *FNDC1* SNPs associated with CHD risk stratified by hypertension vs. non-hypertension and diabetes vs. non-diabetes.

**SNP-ID**	**Model**	**Genotype**	**Hypertension vs. non-hypertension**		**Diabetes vs. non-diabetes**	
			**OR (95% CI)**	***P***	**OR (95% CI)**	***P***
rs420137	Allele	C	1		1	
		G	0.70 (0.55–0.89)	**0.004**	0.97 (0.75–1.26)	0.806
	Co-dominant	CC	1		1	
		GC	0.53 (0.37–0.75)	**<0.001**	1.17 (0.81–1.68)	0.405
		GG	0.58 (0.33–1.03)		0.72 (0.37–1.37)	
	Dominant	CC	1		1	
		GC-GG	0.54 (0.38–0.76)	**<0.001**	1.08 (0.76–1.53)	0.687
	Recessive	CC-GC	1		1	
		GG	0.82 (0.48–1.38)	0.450	0.66 (0.36–1.23)	0.191
	Additive	–	0.68 (0.53–0.87)	**0.002**	0.96 (0.74–1.25)	0.760
rs386360	Allele	A	1		1	
		C	0.71 (0.55–0.90)	**0.005**	0.96 (0.74–1.26)	0.789
	Co-dominant	A/A	1		1	
		C/A	0.59 (0.41–0.83)	**0.003**	1.22 (0.85–1.76)	0.272
		C/C	0.53 (0.30–0.96)		0.62 (0.30–1.25)	
	Dominant	A/A	1		1	
		C/A-C/C	0.58 (0.41–0.81)	**0.001**	0.11 (0.78–1.57)	0.575
	Recessive	A/A-C/A	1		1	
		C/C	0.70 (0.40–1.21)	0.204	0.56 (0.28–1.10)	0.092
	Additive	–	0.67 (0.52–0.87)	**0.002**	0.96 (0.73–1.25)	0.740
rs7763726	Allele	A	1		1	
		G	0.69 (0.54–0.89)	**0.004**	0.89 (0.67–1.17)	0.394
	Co-dominant	A/A	1		1	
		A/G	0.56 (0.40–0.79)	**0.001**	1.21 (1.85–1.73)	0.293
		G/G	0.59 (0.31–1.12)		0.32 (0.12–0.83)	
	Dominant	A/A	1		1	
		G/A-G/G	0.57 (0.41–0.79)	**0.001**	1.05 (0.74–1.48)	0.796
	Recessive	A/A-G/A	1		1	
		G/G	0.77 (0.41–1.43)	0.407	0.29 (0.11–0.75)	**0.011**
	Additive	–	0.67 (0.51–0.87)	**0.002**	0.88 (0.66–1.16)	0.364

CHD, coronary heart disease; SNP, single nucleotide polymorphism; OR, odds ratio; CI, confidence interval. OR (95% CI) values were calculated by logistic regression analysis with adjustments for age, gender, smoking, and drinking. Bold values are statistically significant (*p* < 0.05).

### Haplotype and MDR analyses

**Figure 1:**
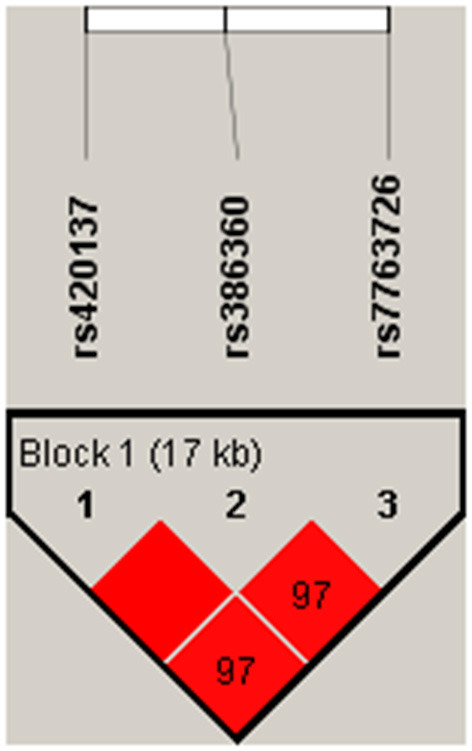
Haplotype analysis was performed to analyze the association of FNDC1.

Haplotype analysis was performed to analyze the association of *FNDC1* polymorphisms with CHD risk, and one block including rs420137, rs386360 and rs7763726 was noted (Figure 1). Meanwhile, the connection between *FNDC1* haplotypes and the risk of CHD is showed in Table 7. We found that the haplotype “GCG” (*p* = 0.002, OR: 1.34, 95% CI: 1.11–1.63) and the haplotype “CAA” (*p* = 0.004, OR: 1.29, 95% CI: 1.09–1.55) had a higher risk of CHD after adjustment.

**Table 7:** Haplotype analysis.

**SNPs**	**Haplotype**	**F-A**	**F-U**	**OR**	***P* ^a^**
rs420137|rs386360|rs7763726	GCG	0.271	0.271	1.34 (1.11–1.63)	**0.002**
	GCA	0.042	0.050	0.82 (0.56–1.21)	0.318
	GAA	0.017	0.014	1.20 (0.62–2.33)	0.588
	CAA	0.340	0.287	1.29 (1.09–1.55)	**0.004**

SNP, single nucleotide polymorphism; F-A, frequency in cases; F-U, frequency in controls; OR, odds ratio; 95% CI, 95% confidence interval.

p^a^ values were calculated using Pearson's chi-squared test. Bold values are statistically significant (*p* < 0.05).

MDR analysis showed that the best three-locus model was rs420137, rs386360, and rs7763726 (Bal. Acc. CV Training: 0.552; Bal. Acc. CV Testing: 0.548; CVC: 10/10; Testing Sensitivity: 0.647; Testing Specificity: 0.397; *p* = 0.0004, OR: 1.52; 95% CI: 1.20–1.92) (Table 8).

**Table 8:** Analysis of SNP-SNP interaction models using MDR method.

**Model**	**Training Bal. Acc**.	**Testing Bal. Acc**.	**CVC**	**OR (95% CI)**	***P***
rs7763726	0.551	0.551	10/10	1.51 (1.20–1.92)	**0.0005**
rs420137,rs7763726	0.551	0.550	9/10	1.51 (1.20–1.92)	**0.0005**
rs420137,rs386360,rs7763726	0.552	0.548	10/10	1.52 (1.20–1.92)	**0.0004**

MDR, multifactor dimensionality reduction; Bal. Acc., balanced accuracy; CVC, cross-validation consistency; OR, odds ratio; 95% CI, 95% confidence interval. *P-*values were calculated using χ^2^ tests. Bold values indicate statistical significance (*p* < 0.05).

## Discussion

CHD is a disease affected by many factors. Diverse environmental and genetic factors have been reported to be associated with the etiology of CHD (14). So far, scores of genetic studies have revealed that genes are related to the pathogenesis of CHD (15). It has been reported that *FNDC1* can activate the cell surface receptor AGS8, regulate the intracellular distribution of vascular endothelial growth factor receptor 2 (VEGFR2), promote VEGF-A-induced vascular endothelial cell signal transduction and growth, promote tumor angiogenesis, and lead to increased tumor stage (16). Hence, we investigated the relationship between *FNDC1* polymorphisms and CHD susceptibility by a case-control study. Our results indicated that *FNDC1*-rs420137, -rs386360, and -rs7763726 played an important role in enhancing the risk of CHD. Subgroup analysis showed that rs420137 increased the susceptibility to CHD in males, patients aged ≤62 years, and smokers. Rs386360 had a higher risk of CHD in males, patients aged ≤62 years, smokers, and non-drinkers. Moreover, the association of rs7763726 with increased CHD susceptibility was also revealed in males, patients aged ≤62 years, smokers, and drinkers. Last but not least, these three SNPs we selected were protective factors against hypertension in CHD individuals.

*FNDC1* contains a major component of the fibronectin domain (17). Recent studies have shown that *FNDC1* is closely linked to the occurrence of multiple illnesses (8, 18–21). It was also found that *FNDC1* took part in VEGF-mediated endothelial cell angiogenesis, especially the distribution and transportation of VEGFR2, which had a strong tyrosine kinase activity and was a firsthand factor influencing angiogenesis (19, 22). The existing studies have also displayed that *FNDC1* is a pathogenic gene of acute otitis media in children, and has a certain correlation with hypertension (8). In one study, the *FNDC1* polymorphism (rs3003174) was associated with coronary artery aneurysm complications in Kawasaki disease (23). Previous study found that the expression of *FNDC1* was up-regulated in cardiomyocytes induced by ischemia or hypoxia (24). Motohiko Sato et al. found *FNDC1* isolated from rat hearts with repeated transient ischemia of collateral circulation may induce cardiomyocyte apoptosis under inflammatory or hypoxic conditions. Hypoxia induced *FNDC1* mRNA expression in rat aortic smooth muscle cells, endothelial cells, or myocardial fibroblasts, suggesting a cardiomyocyte-specific adaptive mechanism including G protein signaling pathway remodeling (25). Bouchareb et al. (26) found that the mutations in the *FNDC1* gene were associated with cardiovascular disease, indicating that *FNDC1* polymorphisms may be involved in the development of CHD.

Sex and age are strong determinants of CHD risk (27), so is smoking (28). According to sex-stratified analysis, we noticed that the three SNPs in this study affected CHD susceptibility only in males, suggesting that there were gender differences in the influence of SNPs on the risk of CHD. At the same time, these three SNPs increased the risk of CHD in patients aged 62 years and younger and smokers. These results are consistent with previous studies indicating that genetic polymorphisms influence the susceptibility to CHD in males or patients aged ≤61 years (29, 30). Zhao et al. (31) found that the morbidity and mortality of cardiovascular diseases were correlated with the degree of blood pressure elevation. In our study, we observed that rs420137, rs386360 and rs7763726 could protect individuals with CHD from hypertension. These results indicate that patients with CHD need individualized treatment.

We need to emphasize that our study still has some limitations. First, our subjects were all Han Chinese. Second, the sample size was too small to validate the results. Third, CHD is a disease affected by a great number of risk factors. Therefore, we cannot completely exclude the potential impact of the other factors on its development.

## Conclusions

In a word, this study indicated that *FNDC1* polymorphisms (rs420137, rs386360, and rs7763726) were related to CHD risk, suggesting that these three variants may be new biomarkers for predicting CHD risk. To the best of our knowledge, this is the first study on the relationship between *FNDC1* gene polymorphisms and CHD, and the specific mechanism of CHD still needs to be further studied.

## Data availability statement

The original contributions presented in the study are included in the article/supplementary material, further inquiries can be directed to the corresponding author/s.

## Ethics statement

This study was reviewed and approved by the Ethics Committee of the First Hospital of Yulin City, and conducted in accordance with the standards of the Declaration of Helsinki. Written informed consent was obtained from each participant included in the study.

## Author contributions

HM contributed to the study design and performed statistical analysis. XH was responsible for manuscript preparation. XL and XD performed experiments. JH was responsible for checking the data. HZ and YZ contributed to data collection. All authors were responsible for drafting the manuscript, they have read, and approved the final version.

## Conflict of interest

The authors declare that the research was conducted in the absence of any commercial or financial relationships that could be construed as a potential conflict of interest.

## Publisher's note

All claims expressed in this article are solely those of the authors and do not necessarily represent those of their affiliated organizations, or those of the publisher, the editors and the reviewers. Any product that may be evaluated in this article, or claim that may be made by its manufacturer, is not guaranteed or endorsed by the publisher.
